# Inside or out? Clonal thiotrophic symbiont populations occupy deep-sea mussel bacteriocytes with pathways connecting to the external environment

**DOI:** 10.1038/s43705-021-00043-x

**Published:** 2021-08-17

**Authors:** Tetsuro Ikuta, Yuka Amari, Akihiro Tame, Yoshihiro Takaki, Miwako Tsuda, Ryo Iizuka, Takashi Funatsu, Takao Yoshida

**Affiliations:** 1 Japan Agency for Marine-Earth Science and Technology (JAMSTEC), 2-15 Natsushima, Yokosuka, Kanagawa 237-0061 Japan; 2 Tokyo University of Marine Science and Technology (TUMSAT), 4-5-7 Konan, Minato-ku, Tokyo, 108-8477 Japan; 3 Marine Works Japan, Ltd., 3-54-1 Oppamahigashi, Yokosuka, Kanagawa 237-0063 Japan; 4 Graduate School of Pharmaceutical Sciences, The University of Tokyo, 7-3-1 Hongo, Bunkyo-ku, Tokyo, 113-0033 Japan; 5 Present Address: Department of Biological Sciences, Graduate School of Science, The University of Tokyo, 7-3-1 Hongo, Bunkyo-ku, Tokyo, 113-0033 Japan

**Keywords:** Symbiosis, Animal physiology, DNA sequencing, Marine microbiology

## Abstract

Deep-sea *Bathymodiolus* mussels are generally thought to harbour chemosynthetic symbiotic bacteria in gill epithelial cells called bacteriocytes. However, previously observed openings at the apical surface of bacteriocytes have not been conclusively explained and investigated as to whether the *Bathymodiolus* symbiosis is intracellular or extracellular. In this study, we show that almost all the membranous chambers encompassing symbionts in a single bacteriocyte of *Bathymodiolus septemdierum* are interconnected and have pathways connecting to the external environment. Furthermore, the symbiont population colonising a single bacteriocyte is mostly clonal. This study hypothesises on a novel model of cellular localization at the interface between extra- and intracellular symbiosis, and the cellular-level process of symbiont acquisition in *Bathymodiolus* mussels.

## Introduction

Mussels of the bivalve family Mytilidae have provided insights into microbial symbiosis and adaptations that arose during the evolution of deep-sea chemosynthetic fauna. Deep-sea mussels often dominate in reducing environments such as hydrothermal vents, methane seeps, whale falls, and sunken wood, and have symbiotic relationships with either or both thiotrophic and methanotrophic bacteria that inhabit their gills [1, 2]. Additionally, they exhibit two modes for the localisation of the symbionts: extracellular and intracellular [1, 2]. Evolutionary scenarios for the transition of symbiont locations from extracellular to intracellular have been discussed previously, and deep-sea mussels belonging to the genus *Bathymodiolus* could have evolved intracellular symbiosis in gill cells called bacteriocytes [1–3]. Symbionts in the vacuoles (symbiosomes) of *Bathymodiolus* bacteriocytes are assumed to be acquired from the environment, and open-pit structures in the cell membranes of the gill surface have been considered as the sites for the endocytosis of free-living bacteria [4]. In contrast, similar openings in *Bathymodiolus* and several symbiotic gastropod species have been interpreted as open connections to the exterior seawater, illustrating a possible evolutionary pathway from extra- to intracellular symbioses [5–7]. Thus, such inconsistencies in interpreting open-pit structures have existed for many years and there is no consensus regarding the extra- or intracellular nature of *Bathymodiolus* symbiosis.

To address this issue, we investigated the detailed structure of bacteriocytes of *Bathymodiolus septemdierum* harbouring a species of the thiotrophic symbiont including different strains with differing metabolic capacities [8], with tomographic three-dimensional analysis of the ultrastructure of membranous symbiotic chambers. Additionally, we analysed the population diversity of the symbionts in a single bacteriocyte.

## Results

**Fig. 1:**
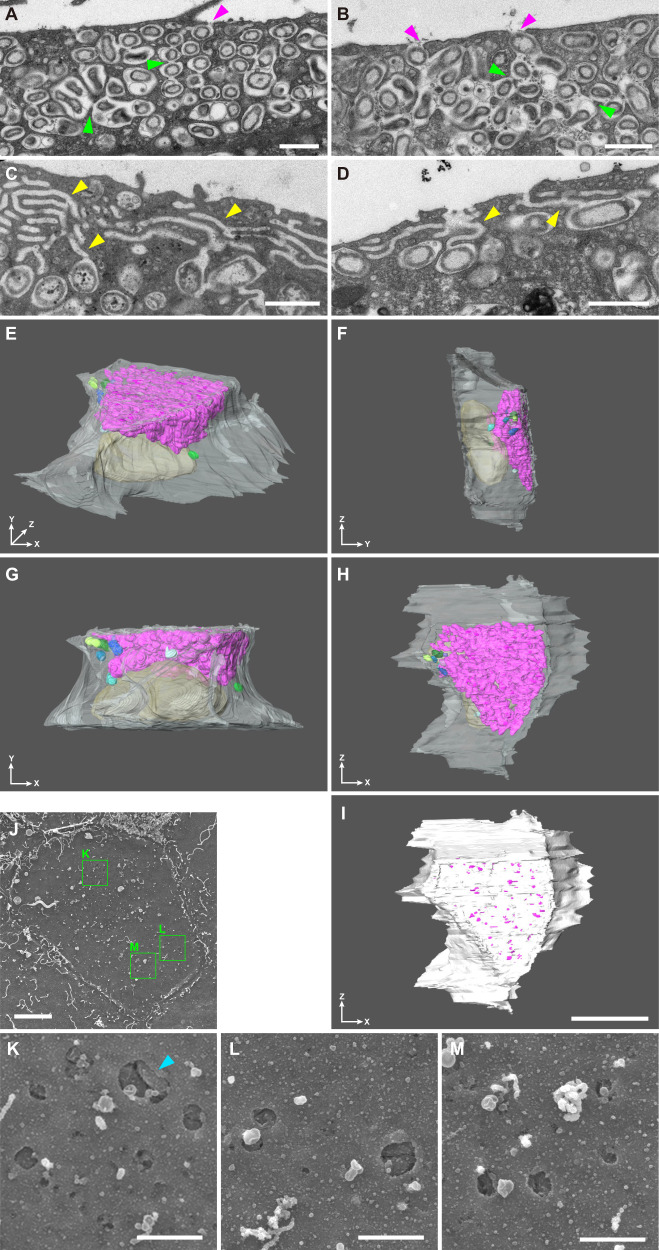
Detailed structure of bacteriocytes of *Bathymodiolus septemdierum*. (A–D) Transmission electron microscopy images showing openings of the symbiotic chambers at the apical surfaces of bacteriocytes (magenta arrowheads in A and B), linkage among several chambers (green arrowheads in A and B), and the complex passages connecting the chambers and the external environment (yellow arrowheads in C and D). Apical is at the top. (E–I) Tomographic three-dimensional reconstruction of bacteriocyte ultrastructure from thin sections of cell 1 observed by scanning electron microscopy (SEM) as a representative showing that almost all symbiotic chambers are interconnected (E–H), with many openings to the external environment at the apical surface (I). Each group of interconnected chambers is labelled with a different colour (the interlinked chambers encompassing the greatest volume are labelled with magenta). (E–H) Views of a single bacteriocyte from four directions, which are indicated at the bottom left of each panel. Because of the resolution of the serial sections (80 nm), several linkages between chambers may have been missed. The nucleus and cytoplasm are labelled with yellow and white, respectively, and made slightly transparent. (I) Apical surface of the 3D reconstructed bacteriocyte. Magenta spots indicate the openings of the chambers. (J–M) SEM images showing many openings at the apical surface of a bacteriocyte of *B. septemdierum*. (K–M) Magnified images of the cell surface in the areas indicated by green rectangles in (J). Note that the symbionts are observed through the openings (light-blue arrowhead). Scale bars in (A–D and K–M) 1 µm; (I and J) 5 µm.

In our transmission electron microscopy observations of *B. septemdierum* gills, the symbiotic chambers at the apical surfaces of bacteriocytes were occasionally open to the exterior environment and interconnected to other chambers located more basally (Fig. 1A, B, and Supplementary Table S1). Additionally, we observed complex passages in several bacteriocytes connecting the symbiotic chambers and the environment at the apical end (Fig. 1C, D). Furthermore, our tomographic three-dimensional reconstruction of the bacteriocyte (cells 1–3) ultrastructure from thin sections revealed that almost all chambers (>98.5% in volume, Supplementary Table S2) were interconnected (Fig. 1E–H, Supplementary Figs. S1, S2, and Supplementary videos), with many openings to the environment at the apical surface (Fig. 1I, Supplementary Fig. S2, and Supplementary Tables S1, S2). Consistent with these observations, scanning electron microscopy also revealed many openings (size average: 296.3 (standard deviation, SD = 214.6; *n* = 30) nm × 214.7 (SD = 163.8; *n* = 30) nm) at the apical surface of bacteriocytes of *B. septemdierum* (Fig. 1J–M); however, these were not observed in a reference species, *Bathymodiolus japonicus*, which harbours a methanotrophic symbiont (Supplementary Fig. S3 and Supplementary Table S1).

**Fig. 2:**
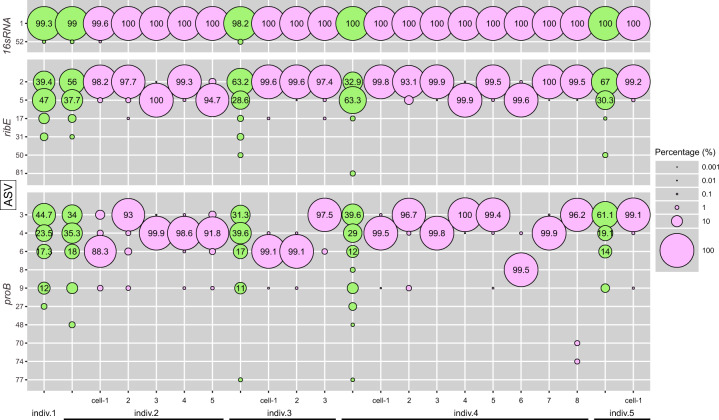
Percentage of amplicon sequence variants (ASVs) for each of three genes, *16* *S rRNA*, *ribE*, and *proB*, detected from gill tissue masses or single bacteriocytes. Nucleotide diversities of the three genes were analysed for each mass of gill tissue from five *Bathymodiolus septemdierum* individuals (indiv. 1–5, light green) and each single bacteriocyte from four individuals (indiv. 2–5, light magenta). Percentages of ASV ≥ 10% are shown in each bubble. Very minor ASVs of a maximum of 1% or less throughout all samples were omitted from the figure, but the data is available in Supplementary Fig. S4 and Supplementary Table S4.

Considering that almost all the symbiotic chambers were linked, we suspected that symbionts within a single bacteriocyte are clones. We conducted an amplicon sequencing experiment of two single-copy symbiont genes, *ribE* and *proB* (Supplementary text and Supplementary Tables S1, S3). More than two major amplicon sequence variants (ASVs) for each gene were detected in each gill tissue mass, whereas only one major ASV was dominating in each single bacteriocyte (Fig. 2, Supplementary Table S4, Supplementary Fig. S4), demonstrating that one bacteriocyte is occupied by almost exclusively one bacterial strain.

## Discussion and conclusion

Our study provides the first evidence that almost all symbiotic chambers in a single bacteriocyte of *B. septemdierum* are interconnected and have multiple pathways connecting to the external environment, occasionally via complex passages. The ‘hollow’ structure (a large space surrounded by pseudopodium-like structures) at the apical surface of gill cells in *Adipicola pacifica* has been described as an intermediate state between extra- and intracellular symbiosis in the deep-sea mussels [2]. However, the unique cell morphology found in *B. septemdierum* has never been reported and may reflect a novel type of intermediate stage between extra- and intracellular symbiosis, namely, a state close to intracellularity but having extracellular properties (Supplementary Fig. S5). The openings of apical vacuoles have been reported mainly for the symbiosomes enclosing thiotrophic bacteria and rarely for those enclosing methanotrophs [4, 5], and in this study, they were not observed in *B. japonicus* harbouring methanotrophic symbiont (Supplementary Fig. S3). *Bathymodiolus* mussels such as *B. septemdierum* and other species with thiotrophic symbionts may possess the morphological properties of extracellular symbiosis while symbiosis with methanotrophic bacteria is truly intracellular. The relationship between the extracellular characteristics found in this study and the high degree of genomic diversity in *Bathymodiolus* thiotrophic symbionts [9] will require further investigation. In *Bathymodiolus* mussels, symbionts may infect the newly formed gill filaments from the environment or more likely via self-infection within a host individual throughout the mussel’s life [9, 10]. The larger openings we observed may function to release the symbiotic bacteria towards the adjacent gill filament. Furthermore, the extended membrane network consisting of interconnected chambers and complex passages in the bacteriocyte may increase the surface area for host uptake of nutrients released by the symbiont. The transfer of chemical substances and symbionts through the apical openings and complex passages also needs to be investigated in the future.

Fluorescence *in situ* hybridisation (FISH) analyses have suggested that individual bacteriocytes are dominated by a single symbiont strain [8, 11]. Our results demonstrated that the symbiont population colonising a single bacteriocyte of *B. septemdierum* is mostly clonal. The minor ASVs detected in each single bacteriocyte could be due to experimental contamination, considering the dissection of the gills and subsequent processes for cell collection, or could be attributed to PCR or sequencing errors (Supplementary Fig. S4). However, we could not completely exclude the possibility that the symbionts hosted in these smaller, discontinued chambers are distinct but very low abundant strains. A variety of symbiotic strains are known to exist in the external environment and in the host individuals [8]. Considering the clear boundaries of patchy staining under FISH analysis [8] and the fact that the symbiont population colonising a single bacteriocyte is almost clonal, it is unlikely that a single bacteriocyte is infected multiple times. Rather, a single bacteriocyte may take up a single symbiont cell only once at the early stage of gill filament formation. The space enclosing the symbiont by the cell membrane may continue to branch out along with proliferation of the symbiont, while maintaining the extracellular connection, occasionally forming new pathways to the exterior environment (Supplementary Fig. S5). Thus, the first strain that colonizes the bacteriocyte would become the dominating strain and prevents other strains from entering the bacteriocyte. This model does not contradict the intermixing concept of the symbiont population at the level of individual hosts [11]. Alternatively, multiple strains can colonise a single bacteriocyte, but symbiont strains with better performance might proliferate more successfully to become the major strain, and outcompete the symbiont with lower performance. The latter may remain present in low abundances in small, isolated chambers. However, since multiple strains are distributed in a mosaic pattern along the gill filament [8], microenvironmental diversity is unlikely to exert selection pressure on the symbiont that affects bacteriocyte colonisation.

This study presents a novel morphology corresponding to an intermediate symbiotic state between extracellular and intracellular location, along with a hypothesis for cellular-level symbiont acquisition processes in *Bathymodiolus* mussels. Information from additional species and taxa is required to test the generality of these hypotheses for symbiont localisation and uptake into individual bacteriocytes.

## Supplementary information

Supplementary material

Supplementary video S1

Supplementary video S2

Supplementary video S3

Supplementary Table S4

## References

[1] Lorion J, Kiel S, Faure B, Kawato M, Ho SY, Marshall B (2013). Adaptive radiation of chemosymbiotic deep-sea mussels. Proc R Soc B.

[2] Fujiwara Y, Kawato M, Noda C, Kinoshita G, Yamanaka T, Fujita Y (2010). Extracellular and mixotrophic symbiosis in the whale-fall mussel *Adipicola pacifica*: a trend in evolution from extra- to intracellular symbiosis. PLoS One.

[3] Miyazaki J, de Oliveira Martins L, Fujita Y, Matsumoto H, Fujiwara Y (2010). Evolutionary process of deep-sea *Bathymodiolus* mussels. PLoS One.

[4] Won YJ, Hallam SJ, O'Mullan GD, Pan IL, Buck KR, Vrijenhoek RC (2003). Environmental acquisition of thiotrophic endosymbionts by deep-sea mussels of the genus *Bathymodiolus*. Appl Environ Microbiol.

[5] Dubilier N, Windoffer R, Giere O (1998). Ultrastructure and stable carbon isotope composition of the hydrothermal vent mussels *Bathymodiolus brevior* and *B*. sp. affinis *brevior* from the North Fiji Basin, western Pacific. Marine Ecology Progress Series.

[6] Endow K, Ohta S (1989). The symbiotic relationship between bacteria and a mesogastropod snail, *Alviniconcha hessleri*, collected from hydrothermal vents of the Mariana Back-Arc Basin. Bulletin of Japanese Society of Microbial Ecology.

[7] Windoffer R, Giere O (1997). Symbiosis of the hydrothermal vent gastropod *Ifremeria nautilei* (Provannidae) with endobacteria - structural analyses and ecological considerations. Biol Bull.

[8] Ikuta T, Takaki Y, Nagai Y, Shimamura S, Tsuda M, Kawagucci S (2016). Heterogeneous composition of key metabolic gene clusters in a vent mussel symbiont population. ISME J.

[9] Romero Picazo D, Dagan T, Ansorge R, Petersen JM, Dubilier N, Kupczok A (2019). Horizontally transmitted symbiont populations in deep-sea mussels are genetically isolated. ISME J.

[10] Wentrup C, Wendeberg A, Schimak M, Borowski C, Dubilier N (2014). Forever competent: deep-sea bivalves are colonized by their chemosynthetic symbionts throughout their lifetime. Environ Microbiol.

[11] Ansorge R, Romano S, Sayavedra L, Porras M, Kupczok A, Tegetmeyer HE (2019). Functional diversity enables multiple symbiont strains to coexist in deep-sea mussels. Nat Microbiol.

