# Supplementary material for: Inside or out? Clonal thiotrophic symbiont populations occupy deep-sea mussel bacteriocytes with pathways connecting to the external environment
Source: ISME Commun. 2021 Aug 17;1:38. doi: 10.1038/s43705-021-00043-x (PMC9723662; doi:10.1038/s43705-021-00043-x)
Supplement: Supplementary file 1 — Supplementary material [file 43705_2021_43_MOESM1_ESM.pdf]

Supplementary materials

Supplementary text

Materials and methods

Supplementary figure legends (Figure S1–5)

Supplementary video descriptions (Video S1–3)

References

Supplementary figures (Figure S1–5)

Supplementary tables* (Table S1–3)

*Supplementary table S4 is provided as a separate Excel file.

Materials and methods

Animal sampling

Bathymodiolus septemdierum were collected from hydrothermal vent sites on the Myojin Knoll, Japan, at depths of 1182 and 1232 m during cruises KY15-07 (24–29 April 2015) and KS-16-5 (10–13 May 2016) with the ROV *Hyper Dolphin* operated by the R/V *Kaiyo* or R/V *Shinsei-Maru* of the Japan Agency of Marine-Earth Science and Technology (JAMSTEC) (Supplementary table S1). *Bathymodiolus japonicus* were collected from the Off Hatsushima Island seep site in Sagami Bay, Japan, at a depth of 908 m during cruise KS-20-1 (7–11 January 2020) with the ROV *Hyper Dolphin* operated by the R/V *Shinsei-*

Maru (Supplementary table S1).

Electron microscopy

The gills of the mussels (shell length: 8–11 cm, Supplementary table S1) were excised onboard, fixed with 2.5% glutaraldehyde in filtered seawater, and stored at 4 °C. Transmission electron microscopy of the gills of three *B. septemdierum* individuals (Supplementary table S1) was performed as previously described [1, 2].

For scanning electron microscopy (SEM) to reconstruct 3D gill cell images, a piece of the stored gill of *B. septemdierum* (Supplementary table S1) was washed in filtered artificial seawater (FSW) and post-fixed with 2% osmium tetroxide dissolved in FSW for 2 h at 4 °C. After washing with 8% sucrose aqueous solution, conductive staining was performed with 0.5% thiocarbohydrazide solution (Thermo Fisher Scientific, Waltham, MA, USA) for 30 min at 4 °C, and with 1% osmium tetroxide aqueous solution for 1 h at 4 °C. After washing with 8% sucrose solution, the sample was dehydrated in an ethanol dilution series, successively immersed in QY1 (Nissin EM) and QY1/Quetol resin (2:1, 1:1, 1:2) mixtures, and finally embedded in Quetol resin. Ultra-thin sections (80 nm thickness) were prepared using an EM-UC7 microtome with a diamond knife (Leica Microsystems, Wetzlar, Germany). A total of 350 serial sections were prepared for one *B. septemdierum* individual (Supplementary table S1), collected on S9445 glass slides

(Matsunami Glass, Osaka, Japan), stained with 2% uranyl acetate aqueous solution, and coated with osmium using an OPC80 osmium plasma coater (Filgen, Aichi, Japan). The sections were observed under an FEI Quanta 450FEG scanning electron microscope (Thermo Fisher Scientific) operated at 5 kV. To cover the entire cell thickness, 145, 239, and 212 serial sections were photographed at 20000 × magnification for each of the three bacteriocytes. In addition, parts of sections were photographed at 55000 × magnification in order to supplement the observation of details.

To observe the apical surface of the gill cells by using SEM, pieces of the gill from three individuals of *B. septemdierum* and *B. japonicus* (Supplementary table S1) were post-fixed with 2% osmium tetroxide in FASW for 2 h. The samples were stained with 0.2% tannic acid in distilled water (DW) for 30 min and 1% osmium tetroxide in DW for 1 h. After dehydration in an ethanol dilution series, the samples were immersed for 10 min in successive mixtures of ethanol/isoamyl acetate (2:1, 1:2), and three times in isoamyl acetate. The samples were dried with a JCPD-5 critical point impression device (JEOL, Tokyo, Japan), coated with osmium using an OPC80 osmium plasma coater, and observed under an FEI Quanta 450FEG electron microscope (Thermo Fisher Scientific) operated at 5 kV.

Tomographic three-dimensional reconstruction of bacteriocyte ultrastructure from

thin sections

Images of serial sections obtained from SEM were imported into AMIRA v3.0 (Thermo Fisher Scientific) and aligned into a single stack. The aligned data were imported into another image analysis software, Microscopy Image Browser (MIB [3]), and materials of interest (symbiotic chamber, cell nucleus, and cell membrane) were manually traced for segmentation throughout the image stack (Supplementary figure S1). After segmentation, the data were returned to AMIRA, and 3D tomographic images were constructed with post-processing, including surface rendering and smoothing. Additionally, the extracted material volume was calculated using the surface area volume function of AMIRA. The raw images for tomographic three-dimensional reconstruction are available from the corresponding author upon reasonable request.

Single gill cell preparation

The gills were excised from the five *B. septemdierum* individuals (Supplementary table S1) using a scalpel cleaned with ethanol. Part of the excised gill was incubated in an autoclaved calcium-and magnesium-free balanced salt solution (CMF) containing 508 mM NaCl, 10 mM KCl, 8.7 mM $NaHCO_3$, 28.6 mM Na_2SO_4, 0.1 mM EGTA, and 4 mM glucose at pH 7.2 [4] for 90 min at 20 °C with shaking at 110 rpm. Subsequently, the gills were treated with 1 mg/mL collagenase type I (Fujifilm Wako Pure Chemical, Osaka,

Japan), 400 U/mL hyaluronidase (Fujifilm Wako Pure Chemical), and 4 mM glucose in autoclaved artificial seawater (ASW) for 90 min at 20 °C. After washing in CMF, the gill was incubated in CMF with 1 mg/mL bovine serum albumin (BSA), 0.4 mg/mL trypsin inhibitor (Fujifilm Wako Pure Chemical), and 3 U/mL recombinant DNaseI (Takara Bio, Shiga, Japan) for 15 min at room temperature, and then placed on ice. The gill was flushed 20–30 times using a siliconised Pasteur pipette to separate the cells. The cell suspensions were filtered through a 41 µm nylon mesh filter and centrifuged at 150 × g for 5 min at 4 °C. Precipitated cells were washed with CMF containing 1 mg BSA and centrifuged at 150 × g for 5 min at 4 °C. This step was repeated twice. Finally, isolated cells were suspended in ASW with 1 mg/mL BSA and 5 µg/mL DAPI and encapsulated in water-in-oil (W/O) microdroplets as follows. W/O microdroplets were prepared using a microfluidic device, and one W/O microdroplet, including a single bacteriocyte showing the symbiont DAPI signals within the cell by fluorescence microscopy, was transferred onto the lid of a 0.2 mL PCR tube, as described previously [5]. Each isolated cell was recovered in 5 µL of nuclease-free water (Thermo Fisher Scientific) in a PCR tube by centrifugation and stored at −80 °C until use.

Diversity analysis of symbiotic bacteria in single bacteriocyte

To identify and select genes containing many single- or multi-nucleotide variant

(SNV/MNV) suitable for strain-discriminative amplicon sequencing, whole genome nucleotide diversity analysis was performed using CLC Genomics Workbench 12.0.3 (Qiagen, Hilden, Germany). Prior to mapping, the Illumina reads (150 bp × 2) of the *B. septemdierum* symbiont genome [6] were trimmed to remove low-quality sequences (Phred score < 20) at the end of reads or adaptor contamination using Trimmomatic v0.36 [7], and those shorter than 100 bp in length were removed. Mapping to the *B. septemdierum* symbiont genome (accession no. AP013042.1) was performed using a mapper implemented in CLC Genomics Workbench with default alignment settings, except for the length fraction = 0.9 and similarity fraction = 0.9. SNV/MNV detection was performed using the 'low frequency variant detection' module with a 1% cut-off for significance (this parameter determines the cut-off value for the statistical test to ensure that a variant did not occur due to sequencing errors) [8].

ribE, encoding riboflavin synthetase, and *proB*, encoding glutamate 5-kinase were single-copy genes in the symbiont genome, with high nucleotide diversity (Pi) [9] values of 0.0143 and 0.0120, respectively, and specific PCR primers were designed for these genes. Additionally, we used the 515F-806R bacterial/archaeal universal primer pair for the *16S rRNA* gene [10] to check the species or ribotype variety in the samples. The sequences of the primers with adaptors for Illumina sequencing were as follows: ribE_F-P5, 5′-ACACTCTTTCCCTACACGACGCTCTTCCGATCTCATCGGTCAGGTTAAAATC-

3′; ribE_R-P7, 5′-GTGACTGGAGTTCAGACGTGTGCTCTTCCGATCTTCAAACTCACACCATTCAC-3′; proB_F-P5, 5′-ACACTCTTTCCCTACACGACGCTCTTCCGATCTGAAACCTATGAAACCTTATTTG-3′; proB_R-P7, 5′-GTGACTGGAGTTCAGACGTGTGCTCTTCCGATCTCCTGTTCTACTGGCAACTT-3′; 515F-P5, 5′-ACACTCTTTCCCTACACGACGCTCTTCCGATCTGTGCCAGCMGCCGCGGTAA-3′; and 806R-P7, 5′-GTGACTGGAGTTCAGACGTGTGCTCTTCCGATCTGGACTACHVGGGTWTCTAAT-3′. The *ribE*, *proB*, and *16S rRNA* PCR amplicon lengths, excluding the adapter sequences, were 416, 365, and 292 bp, respectively, and contained 17, 12, and 0 SNV/MNV sites in the symbiont genome, respectively. The average allele frequencies of the SNV/MNV sites in the target range of PCR for *ribE* and *proB* were 37.9% and 38.6%, respectively (Supplementary Table S3).

Multiplex PCR was performed using primer sets for *ribE*, *proB,* and *16S rRNA* genes in 25 µL reaction mixtures containing 12.5 µL of Ampdirect Plus (Shimadzu, Kyoto, Japan), 2 µM of primer set for *16S rRNA*, 0.2 µM of the primer sets for *ribE* and *proB*, 0.625 U of BIOTAQ HS DNA polymerase (Meridian Bioscience, Cincinnati, OH, USA), and 5 µL of a single bacteriocyte with nuclease-free water or 0.01 ng of the genomic DNA

extracted from 30 mg of the gills (corresponding to several gill filaments) of the same individuals that was used to prepare the single bacteriocyte suspension. The reaction consisted of 10 min at 95 °C and 40 cycles of 94 °C for 30 s, 52 °C for 30 s, and 72 °C for 20 s. Amplification of the three genes was confirmed by electrophoresis, and the amplified products were subjected to a second round of PCR after purification using the ExoSAP-IT PCR Product Cleanup Reagent (Affymetrix, CA, USA). The second amplification was performed to add multiplexing indices and Illumina sequencing adapters using Ex Taq polymerase (Takara Bio). The reaction consisted of 1 min at 96 °C, 14 cycles of 96 °C for 30 s, 65 °C for 45 s, and 72 °C for 1 min, followed by 7 min of incubation at 72°C. The adapter-attached PCR products were purified with Agencourt AMPure XP (Beckman Coulter, CA, USA) and quantified using a Qubit 1X dsDNA HS Assay Kit (Thermo Fisher Scientific). The quality and concentration of the PCR products were checked using an Agilent 2100 Bioanalyzer (Agilent Technologies, CA, USA) and real-time PCR with the KAPA Library Quantification Kit (Kapa Biosystems, MA, USA). Sequencing was performed on a MiSeq system (Illumina, CA, USA) using the MiSeq reagent kit v3, as recommended by the manufacturer, and yielded an average of 0.14M paired-end reads per sample, which were deposited into the DDBJ database under the accession number DRA011658.

Amplicon sequence data were processed by merging raw paired-end reads using PEAR v0.9.10 [11], and removing primer sequences using Cutadapt v1.10 [12]. Low-

quality (Q score < 30 in over 3% of sequences) and short (<150 bp) reads were filtered out using a custom Perl script. The resulting sequences were processed to generate a table of amplicon sequence variants (ASVs) using DADA2 v1.14.1 [13] with default parameters. Graphics were produced using either the R base package or ggplot2 package [14].

Supplementary figure legends

Figure S1 Segmentation on the representative sections observed by SEM for tomographic three-dimensional reconstruction of bacteriocyte ultrastructure. (A) cell 1, (B) cell 2, and (C) cell 3. The interlinked chambers encompassing the greatest volume, nucleus, and cytoplasm are labelled with magenta, yellow, and white, respectively, and made slightly transparent. Apical is at the top. Scale bars represent 2 μm.

Figure S2 Tomographic three-dimensional reconstruction of bacteriocyte ultrastructure from thin sections of (A) cell 2 and (B) cell 3. The viewing directions are indicated at the bottom left of each panel. The symbiotic chambers in each linkage are labelled with different colours. Nucleus and cytoplasm are labelled with yellow and white, respectively, and made slightly transparent, except for each bottom right panel in (A) and (B), which shows the apical surface of the 3D reconstructed bacteriocyte. Scale bars

represent 5 μm.

Figure S3 Scanning electron microscopy images of the apical surface of bacteriocyte of *B. japonicus*. (B–D) Magnified images in the areas indicated by green rectangles in (A). Scale bars in (A) 5 μm; (B–D) 1 μm.

Figure S4 Nucleotide sequences of ASVs of (A) *ribE* and (B) *proB*. The sequences of all ASVs are shown, including very minor ASVs (indicated by asterisks) with a maximum of 1% or less throughout the samples, which are not shown in Fig. 2. The nucleotide variations of ASVs were almost identical to SNV/MNVs extracted from the symbiont genome (indicated by red boxes). Red numbers on the boxes indicate the positions in the genes. Very minor ASVs with asterisks appeared only once in one tissue sample or cell (see Table S4), and those containing nucleotide variations that do not match SNV/MNVs might be due to PCR or sequencing errors.

Figure S5 Hypothetical models at the interface between extra- and intracellular symbiosis (vertical axis: A), and for cellular level process of symbiont acquisition in *Bathymodiolus septemdierum* (horizontal axis: B). (A) In the top panel, symbionts are extracellularly localised in the 'hollow' structure, as observed in *Adipicola pacifica* [15].

The middle panel shows the novel intermediate phase proposed in this study; symbionts are localised in membranous chambers connected to each other and the external environment, occasionally via complex passages as observed in *B. septemdierum*. The bottom panel shows the complete intracellular symbiosis as in *B. japonicus* with methanotrophic bacteria. Evolutionarily, it could go either direction, upward or downward. (B) A single bacteriocyte uptakes a single symbiont cell only once at the early stage of the gill filament formation. The space enclosing the symbiont by the cell membrane continues to branch out along with the proliferation of the symbiont while maintaining the connection to the outside of the bacteriocyte, occasionally forming a new pathway to the environment.

Supplementary video descriptions

Supplementary video S1 Tomographic three-dimensional reconstruction of the bacteriocyte ultrastructure from thin sections of cell 1.

Supplementary video S2 Tomographic three-dimensional reconstruction of the bacteriocyte ultrastructure from thin sections of cell 2.

Supplementary video S3 Tomographic three-dimensional reconstruction of the bacteriocyte ultrastructure from thin sections of cell 3.

References

1. Ikuta T, *et al.* Surfing the vegetal pole in a small population: extracellular vertical transmission of an 'intracellular' deep-sea clam symbiont. *R Soc Open Sci* 2016; **3**: 160130.
2. Ikuta T, *et al.* Identification of cells expressing two peptidoglycan recognition proteins in the gill of the vent mussel, *Bathymodiolus septemdierum*. *Fish Shellfish Immunol* 2019; **93**: 815–822.
3. Belevich I, Joensuu M, Kumar D, Vihinen H, Jokitalo E. Microscopy Image Browser: a platform for segmentation and analysis of multidimensional datasets. *PLoS Biol* 2016; **14**: e1002340.
4. Hand SC. Trophosome ultrastructure and the characterization of isolated bacteriocytes from invertebrate-sulfur bacteria symbioses. *Biol Bull* 1987; **173**: 260–276.
5. Nakamura K, *et al.* Culture-independent method for identification of microbial enzyme-encoding genes by activity-based single-cell sequencing using a water-in-oil microdroplet platform. *Sci Rep* 2016; **6**: 22259.

6. Ikuta T, *et al.* Heterogeneous composition of key metabolic gene clusters in a vent mussel symbiont population. *ISME J* 2016; **10**: 990–1001.

7. Bolger AM, Lohse M, Usadel B. Trimmomatic: a flexible trimmer for Illumina sequence data. *Bioinformatics* 2014; **30**: 2114–2120.

8. Berg MG, *et al.* A Pan-HIV strategy for complete genome sequencing. *J Clin Microbiol* 2016; **54**: 868–882.

9. Nei M. *Molecular evolutionary genetics*. Columbia University Press: New York, 1987.

10. Caporaso JG, *et al.* Global patterns of 16S rRNA diversity at a depth of millions of sequences per sample. *PNAS* 2011; **108**: 4516–4522.

11. Zhang JJ, Kobert K, Flouri T, Stamatakis A. PEAR: a fast and accurate Illumina Paired-End reAd mergeR. *Bioinformatics* 2014; **30**: 614–620.

12. Martin M. Cutadapt removes adapter sequences from high-throughput sequencing reads. *EMBnet J* 2011; **17**: 10–12.

13. Callahan BJ, *et al.* DADA2: High-resolution sample inference from Illumina amplicon data. *Nature Methods* 2016; **13**: 581–583.

14. Wickham H, Sievert C. *Ggplot2 : elegant graphics for data analysis*, 2nd edn. Springer: Houston, Texas, 2016.

15. Fujiwara Y, *et al.* Extracellular and mixotrophic symbiosis in the whale-fall mussel *Adipicola pacifica*: a trend in evolution from extra- to intracellular

symbiosis. *PLoS One* 2010; **5**: e11808.

Figure S1

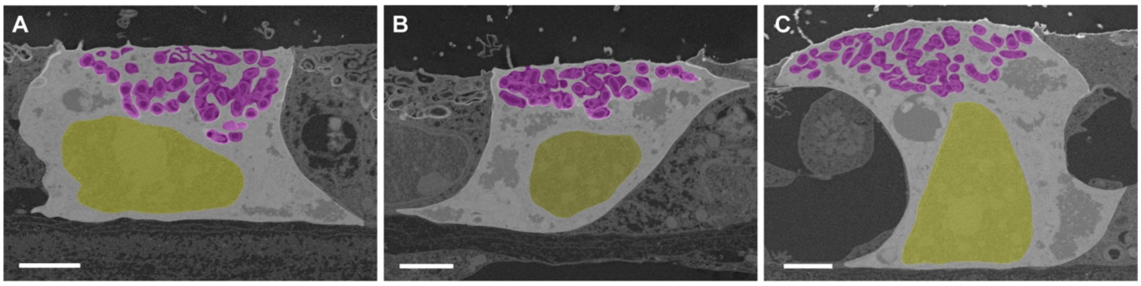

Figure S2

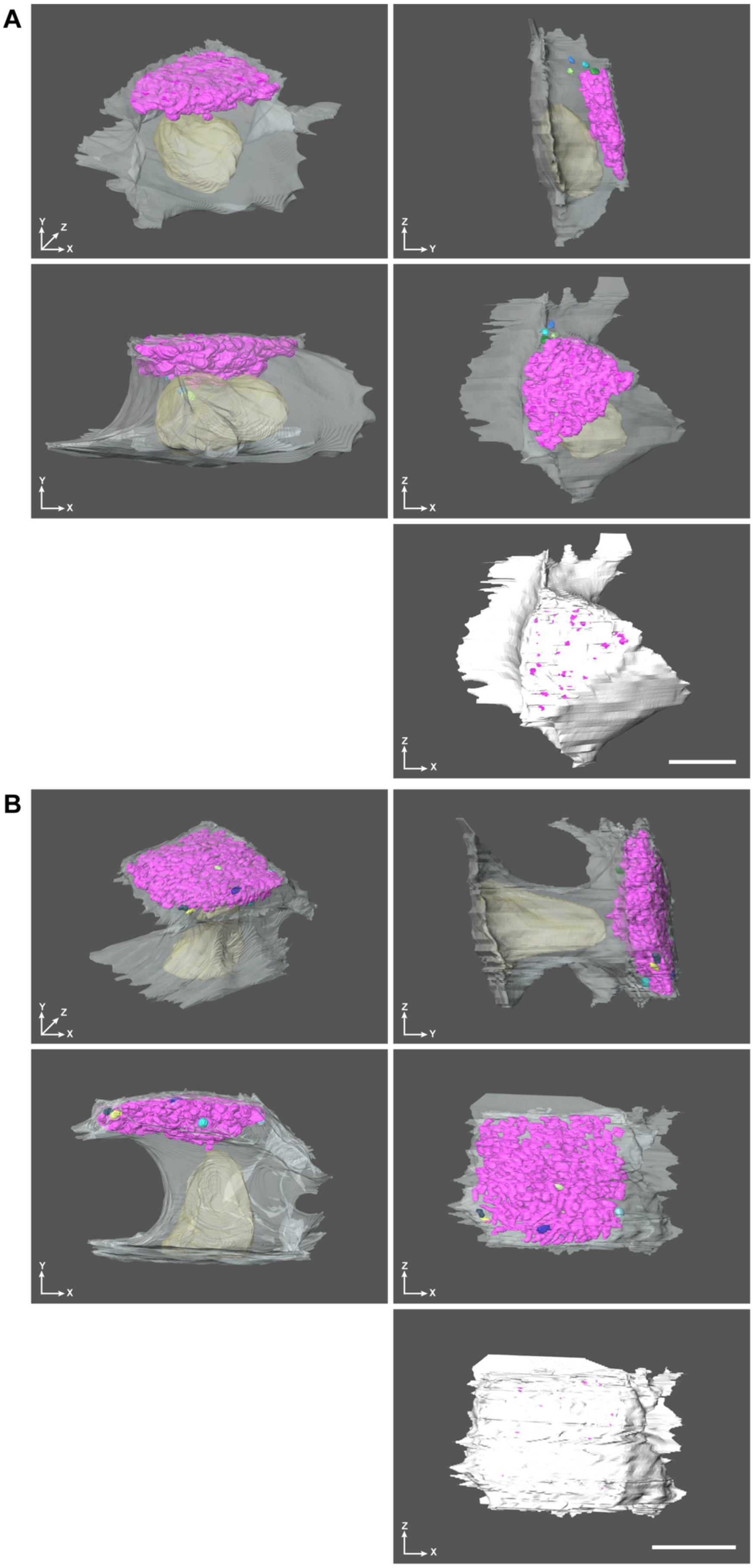

Figure S3

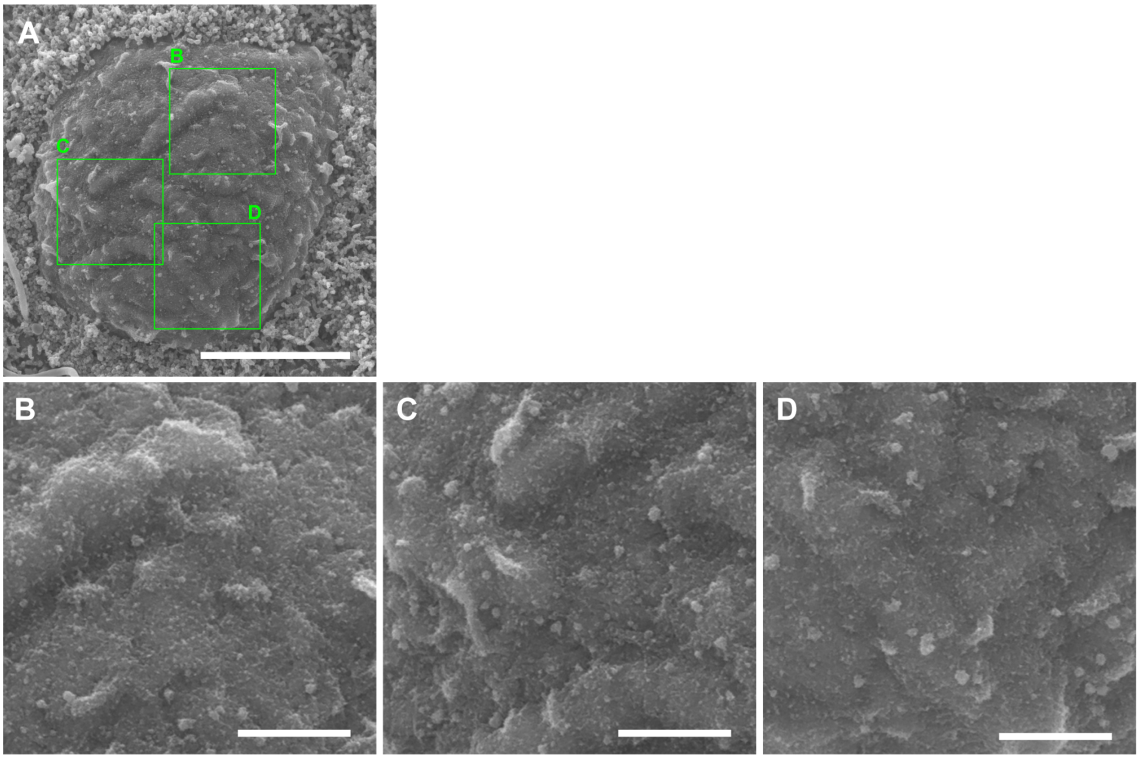

Figure S4 A

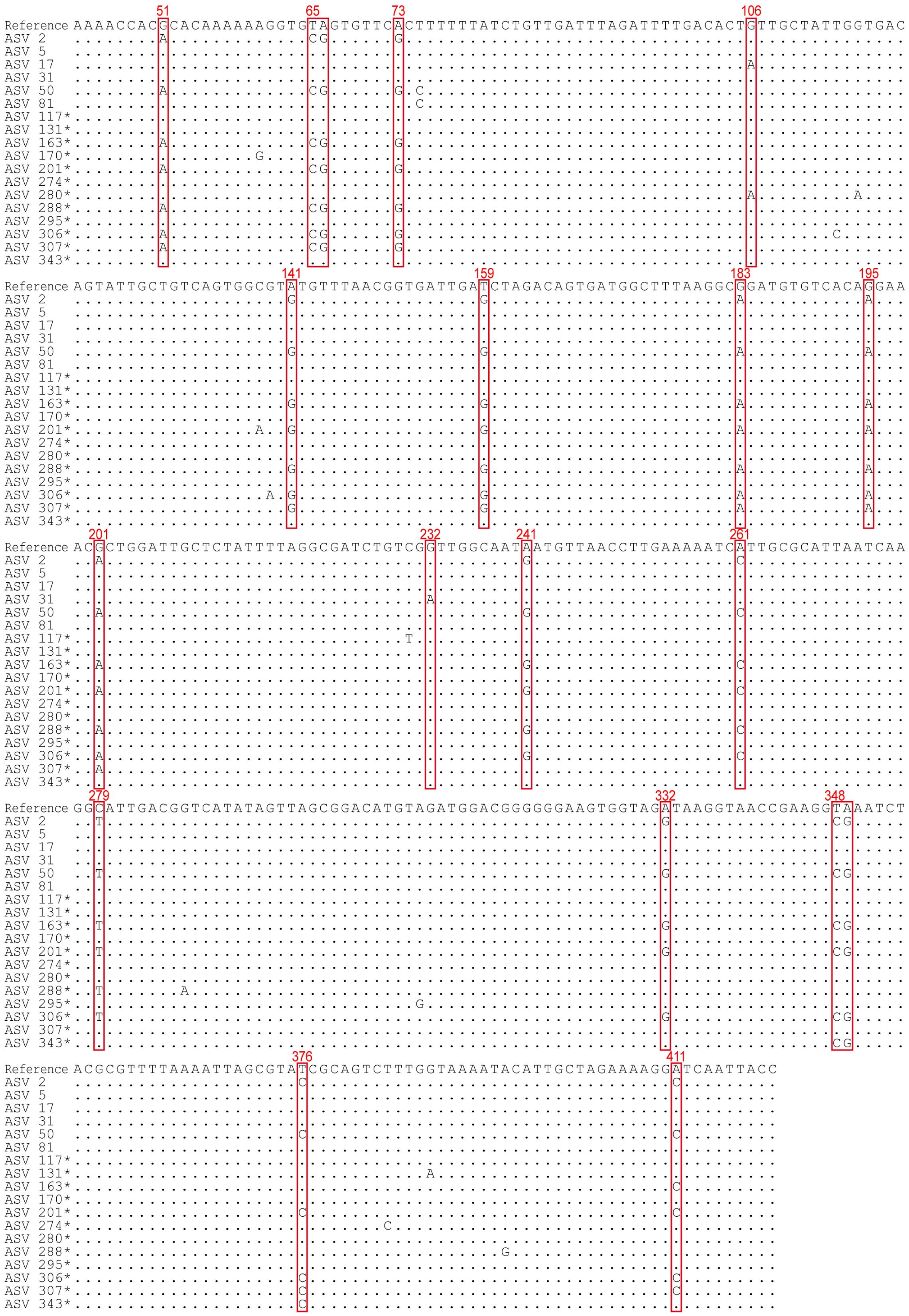
51
65
73
106
Reference AAAACCACGCACAAAAAAGGTGTAGTGTTCACTTTTTTATCTGTTGATTTAGATTTTGACACTGTTGCTATTGGTGAC
141
159
183
195
Reference AGTATTGCTGTCAGTGGCGTATGTTTAACGGTGATTGATCTAGACAGTGATGGCTTTAAGGCGGATGTGTCACAGGAA
201
232
241
261
Reference ACGCTGGATTGCTCTATTTTAGGCGATCTGTCGGTTGGCAATAATGTTAACCTTGAAAAATCATTGCGCATTAATCAA
279
332
348
Reference GGCATTGACGGTCATATAGTTAGCGGACATGTAGATGGACGGGGGGAAGTGGTAGATAAGGTAACCGAAGGTAAATCT
376
411
Reference ACGCGTTTTAAAATTAGCGTATCGCAGTCTTTGGTAAAATACATTGCTAGAAAAGGATCAATTACC
ASV 2
ASV 5
ASV 17
ASV 31
ASV 50
ASV 81
ASV 117*
ASV 131*
ASV 163*
ASV 170*
ASV 201*
ASV 274*
ASV 280*
ASV 288*
ASV 295*
ASV 306*
ASV 307*
ASV 343*

Figure S4 B

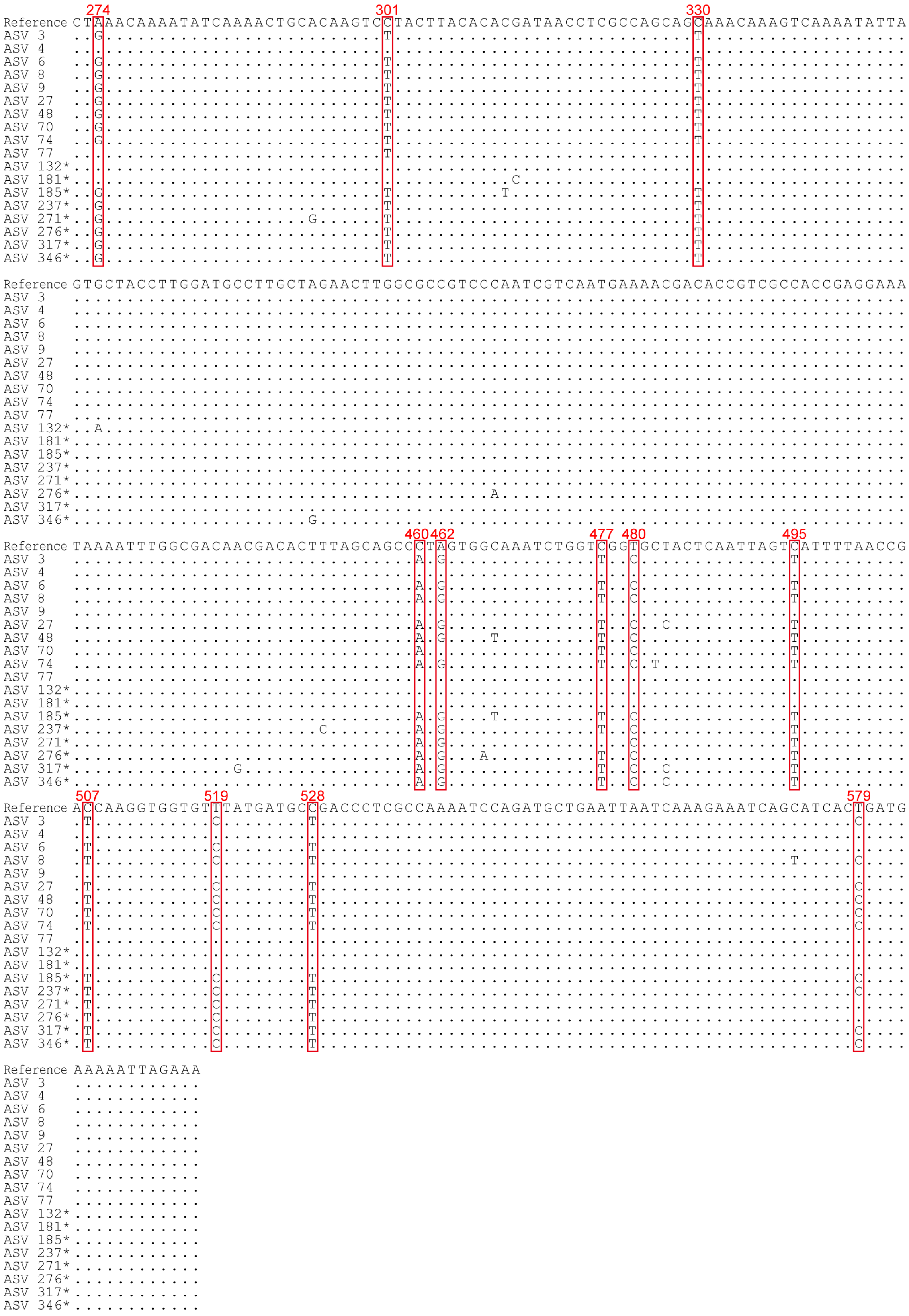
274
301
330
Reference CTAAACAAAATATCAAAACTGCACAAGTCCTACTTACACACGATAACCTCGCCAGCAGCAAACAAAGTCAAAATATTA
Reference GTGCTACCTTGGATGCCTTGCTAGAACTTGGCGCCGTCCCAATCGTCAATGAAAACGACACCGTCGCCACCGAGGAAA
460
462
477
480
495
Reference TAAAATTTGGCGACAACGACACTTTAGCAGCCCTAGTGGCAAATCTGGTCGGTGCTACTCAATTAGTCATTTTAACCG
507
519
528
579
Reference ACCAAGGTGGTGTTTATGATGCCGACCCTCGCCAAAATCCAGATGCTGAATTAATCAAAGAAATCAGCATCACTGATG
Reference AAAAATTAGAAA
ASV 3
ASV 4
ASV 6
ASV 8
ASV 9
ASV 27
ASV 48
ASV 70
ASV 74
ASV 77
ASV 132*
ASV 181*
ASV 185*
ASV 237*
ASV 271*
ASV 276*
ASV 317*
ASV 346*

Figure S5

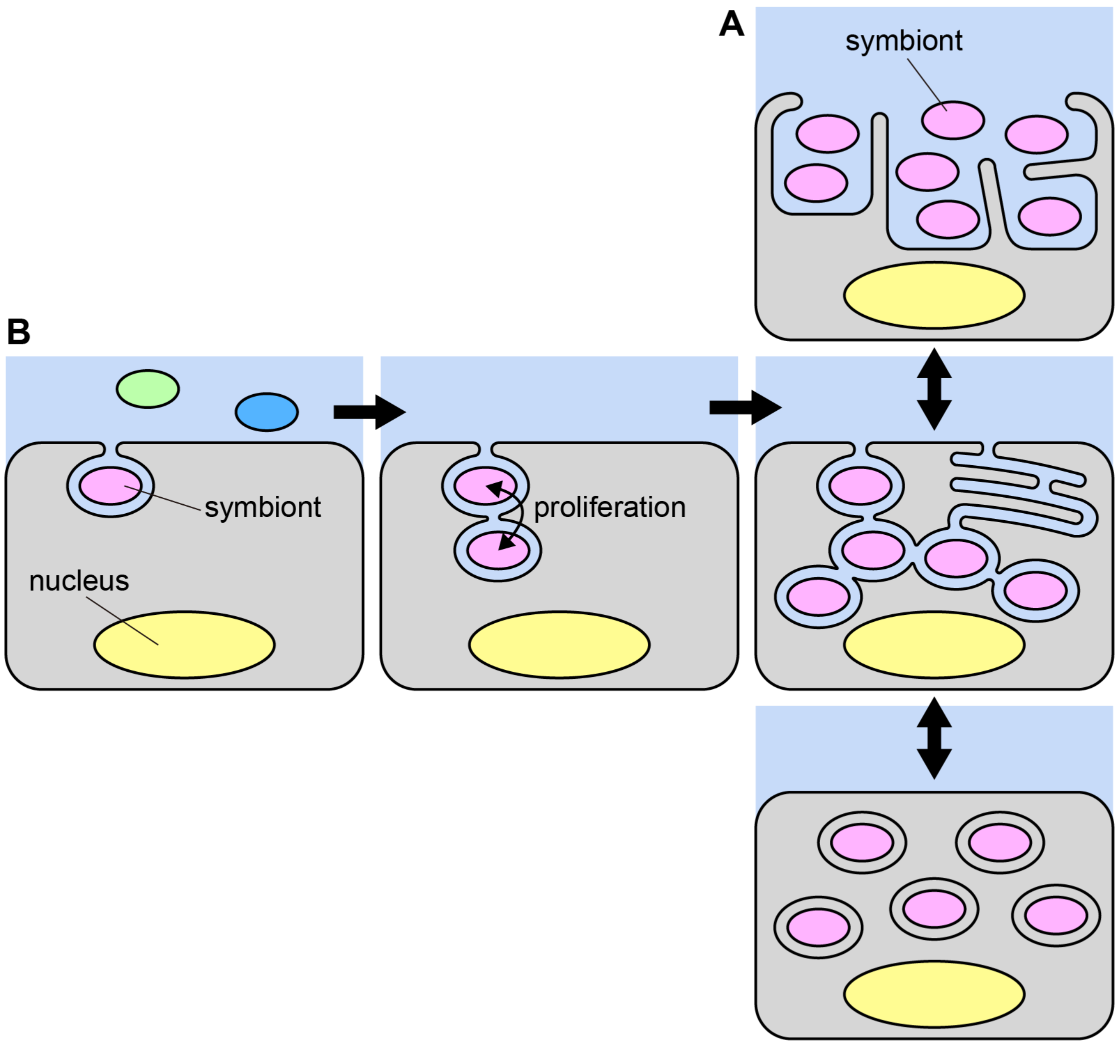

Table S1. Mussel individuals used in this study

Species	Individual ID	Cruise	Shell length (mm)	Experiments	Figure
B. septemdierum	Bs1	KY15-07	109.6	TEM, 3D*, SEM**	1B, E–I, J–M; S1, S2
	Bs2	KY15-07	99.7	TEM, SEM	1C, D
	Bs3	KY15-07	103.4	TEM, SEM	1A
	1	KS-16-5	86.5	ASV†	2
	2	KS-16-5	96.1	ASV	2
	3	KS-16-5	88.1	ASV	2
	4	KS-16-5	98.0	ASV	2
	5	KS-16-5	87.8	ASV	2
B. japonicus	Bj1	KS-20-1	104.2	SEM	S3
	Bj2	KS-20-1	99.1	SEM	–
	Bj3	KS-20-1	96.6	SEM	–

* Tomographic three-dimensional reconstruction of sequential gill sections

** Scanning electron microscopy of the apical surface of bacteriocyte

† Amplicon sequence variant analysis

Table S2: Features of 3D reconstruction of *Bathymodiolus septemdierum* bacteriocytes

	cell 1	cell 2	cell 3
Number of slices for 3D reconstruction	239	212	145
Number of apical openings	45	29	16
Cell volume (μm^3)	375.5	348.4	425.9
Total volume of symbiotic chambers (μm^3): S	50.63	41.12	63.49
Linked chambers with greatest volume (μm^3): L	49.85	40.77	62.83
L/S (%)	98.46	99.15	98.95

Table S3. Single nucleotide variant (SNV)/multi nucleotide variant (MNV) sites in the target range of PCR for *ribE* and *proB*

Gene	Position in the genome	Direction	Position in gene	Type	Reference	Allele	Allele frequency	Average
ribE	903501	–	51	SNV	G	A	45.7	37.9
	903486..903487		65..66	MNV	TA	CG	44.7	
	903479		73	SNV	A	G	45.3	
	903446		106	SNV	G	A	5.6	
	903411		141	SNV	A	G	44.0	
	903393		159	SNV	T	G	44.4	
	903369		183	SNV	G	A	42.2	
	903357		195	SNV	G	A	43.8	
	903351		201	SNV	G	A	43.5	
	903320		232	SNV	G	A	1.1	
	903311		241	SNV	A	G	42.5	
	903291		261	SNV	A	C	43.6	
	903273		279	SNV	C	T	40.5	
	903220		332	SNV	A	G	40.3	
	903203..903204		348..349	MNV	TA	CG	34.8	
	903176		376	SNV	T	C	40.2	
	903141		411	SNV	A	C	42.1	
proB	714708	+	274	SNV	A	G	40.8	38.6
	714735		301	SNV	C	T	40.6	
	714764		330	SNV	C	T	40.3	
	714894		460	SNV	C	A	36.9	
	714896		462	SNV	A	G	37.1	
	714911		477	SNV	C	T	37.4	
	714914		480	SNV	T	C	38.6	
	714929		495	SNV	C	T	40.5	
	714941		507	SNV	C	T	39.2	
	714953		519	SNV	T	C	39.7	
	714962		528	SNV	C	T	39.2	
	715013		579	SNV	T	C	32.3	
